# Refractory facial pain attributed to auriculotemporal neuralgia

**DOI:** 10.1007/s10194-012-0439-4

**Published:** 2012-03-30

**Authors:** Juliana Stuginski-Barbosa, Rafael Akira Murayama, Paulo Cesar Rodrigues Conti, José Geraldo Speciali

**Affiliations:** 1 Bauru School of Dentistry, University of Sao Paulo, Bauru, SP Brazil; 2 Medicine School, University of Sao Camilo, São Paulo, SP Brazil; 3 Faculty of Medicine of Ribeirao Preto, University of Sao Paulo, Ribeirao Preto, SP Brazil

## Introduction

One of the biggest challenge for the clinician is when the patient still persists with complaints of orofacial pain, even with the adoption of well known and appropriate treatment. One of the reasons for this fact can be the misdiagnosis, very often in the field of orofacial pain, since the trigeminal system is frequently influenced by a diversity of different neural inputs. The presence of systemic diseases affecting the masticatory apparatus is also part of this scenario. One of this is a rare condition: auriculotemporal neuralgia (AN) [1].

The aim of the present study was to report a case of refractory facial pain after successful temporomandibular disorder (TMD) management, attributed to AN.

## Case report

A 43-year-old Caucasian female patient presented for treatment of facial pain, with complaint of severe episodic pain in right face, ear and neck, first appeared 12 years ago, worsening in the last 3 months, with crisis of sharp and severe pain. The patient was previously diagnosed with sleep bruxism, depression and insomnia.

Physical examination revealed moderate pain upon palpation of right temporomandibular joint (TMJ), superficial masseter, occipital and sternocleidomastoid muscles. A trigger point was found in right medium masseter muscle referring pain to the ipsilateral ear and TMJ. The maximum mouth opening (MMO) with pain was 39 mm and no other significant signs were detected.

Masticatory myofascial pain and cervicalgia were the initial diagnosis and treatment consisted of advisement of the condition, counseling to avoid clenching her teeth during the day, hot packets and the nocturnal use of an occlusal stabilization splint in the upper jaw. The patient was also referred to a psychologist, physician and physical therapist for management of depression, insomnia and cervicalgia.

After 3 months, the patient reported a significant improvement, with no pain upon muscle palpation or function, and the MMO was 46 mm. However, she complained of a paroxysmal, short-duration pain below the right TMJ and in the temporal region, triggered by MMO and mastication. Intraoral and radiographic exams were unremarkable. Extra oral physical examination revealed that the palpation of the right auriculotemporal nerve region elicited a sharp pain familiar to the patient, which extended from below TMJ to the temporal region.

The hypotheses diagnosis was AN. The auriculotemporal nerve was then blocked with 0.5 ml 2 % lidocaine and 0.5 ml of a suspension containing dexamethasone disodium sulfate (2 mg/ml) and dexamethasone acetate (8 mg/ml) as follows: the needle is inserted below the TMJ, in the posterior margin of the head of the mandible immediately in front of the tragus, to a depth of 1–1.2 cm, at a horizontal 45º angle in the direction of the nose, with care taken to first perform aspiration in order to avoid intravascular injection [1].

There was full improvement of painful symptoms after a single blockade, with no recurrence after a 12-month follow-up.

## Discussion

The diagnosis of orofacial pain is a challenge for dentists and physicians and becomes even more complicated when the signs and symptoms of the patient are due to rare etiologies such AN.

Auriculotemporal neuralgia is characterized by crises of strictly unilateral lancinating pain that may be perceived in the temporal region, TMJ, and in the parotid, auricular and retro-orbital region [2]. In the differential diagnosis, other diseases that might provoke similar AN symptoms must be excluded, such as cervicalgia, TMD, migraine, continuous hemicrania, otitis, trigeminal neuralgia, atypical facial pain, “red ear syndrome”, affection of the parotid gland, temporal arteritis and odontalgia. In this case, an interdisciplinary treatment planning between different health professions was needed for the pain control. Moreover, a conservative, reversible, low-cost and low-tech approach was sufficient for controlling the TMD pain as has been shown previously [3].

According to the International Classification of Headaches, damage to and imprisonment of the peripheral branch of the trigeminal nerve may trigger referred pain in the area innervated by the affected branch [4]. Nerve entrapments in the infratemporal fossa with a spastic condition of the lateral pterygoid muscle may be causally related to compression of auriculotemporal nerve that leads to numbness, pain or both, in the respective areas of nerve distribution [5].

The main clinical characteristics of AN are moderate to severe pain, associated with exacerbations, perceived as stabbing pain. The pain may worsen or be triggered by pressure on the periauricular region at the level of the tragus [1].

In this case, the pain could be triggered with chewing. The lateral pterygoid muscle is activated in mastication. Its superior head contracts during jaw closing while the inferior head contracts during protraction, opening and shifting of the jaw to one side. If either or both of its heads are actively recruited during mandibular closure, this muscle could present an tonic contraction [6] which could lead to stiffness and pain, symptoms commonly found in TMD patients. It is interesting to note that in this case, the relationship between the initial painful symptoms and the final diagnosis of AN is not clear. One hypothesis would be that the patient already had both AN and TMD but, at that moment, the TMD symptoms were possibly more noticeable.

Auriculotemporal neuralgia occurs at a frequency of 0.4 % at a tertiary headache outpatient clinic [1]. However, this frequency may be even higher in outpatient orofacial pain due to the possible involvement of lateral pterygoid muscle in the etiology of auriculotemporal nerve entrapment. It is important to notice that there is no evidence that AN was the cause or consequence of TMD in this case. So far, no data on this were published.

The blockade of the auriculotemporal nerve in the infratemporal fossa is diagnostic and therapeutic. As in others peripheral nerve blocks, it can be achieved with a solution of lidocaine or bupivacaine, with or without a steroid [7].

It is interesting to note that pain relief exceeds the effect of local anesthetic solution in blockades performed not only in AN but also in other peripheral nerve blocks. The use of lidocaine as an anesthetic in these cases appears to promote reversible inhibition of sodium channels, inhibiting nerve conduction in sensory fibers and thus providing temporary analgesia [8].

It has been suggested that chronic changes in nociceptive processing and neuroplasticity may occur in blockades of the occipital nerves, which also has prolonged effect [9]. However, the mechanisms involved in the prolonged effect of peripheral blockade have not been fully elucidated.

Although the effects of locally applied corticosteroids have not been determined, it has been suggested that this treatment may suppress the afferent ectopic firings at the site of nerve injury by an action of C fibers (nociceptive), reducing the release of neuropeptides [10]. The use of prolonged-action corticosteroids in combination with local anesthetics may cause a sufficient prolongation of the time of analgesia to inhibit already established secondary sensitizations, with consequent permanent or greatly prolonged analgesia, as was the case for the present patient.

The present clinical case report suggests that AN may be considered as source of persistent pain in TMD patients and, therefore should be considered in the differential diagnosis or as a comorbid condition, as in the case presented.
